# Synthesis and characterization of methoxy- or cyano-substituted thiophene/phenylene co-oligomers for lasing application†

**DOI:** 10.1039/d0ra04742b

**Published:** 2020-06-23

**Authors:** Takumi Matsuo, Carina Rössiger, Jasmin Herr, Richard Göttlich, Derck Schlettwein, Hitoshi Mizuno, Fumio Sasaki, Hisao Yanagi

**Affiliations:** Graduate School of Science and Technology, Nara Institute of Science and Technology 8916-5 Takayama Ikoma Nara 630-0192 Japan matsuo.takumi.mp2@ms.naist.jp; Institute of Organic Chemistry, Justus-Liebig-University Giessen Heinrich-Buff-Ring 17 35392 Giessen Germany; Institute of Applied Physics, Justus-Liebig-University Giessen Heinrich-Buff-Ring 16 35392 Giessen Germany; Electronics and Photonics Research Institute, National Institute of Advanced Industrial Science and Technology (AIST) 1-1-1 Umezono Tsukuba Ibaraki 305-8568 Japan

## Abstract

As new candidates of thiophene/phenylene co-oligomer (TPCO) species, 5,5′′-bis(4′-methoxy-[1,1′-biphenyl]-4-yl)-2,2′:5′,2′′-terthiophene (BP3T-OMe) and 4′,4′′′-([2,2′:5′,2′′-terthiophene]-5,5′′-diyl)bis(([1,1′-biphenyl]-4-carbonitrile)) (BP3T-CN) were synthesized for lasing applications. Although most unsubstituted TPCO species crystallize in monoclinic form, BP3T-OMe and BP3T-CN crystallized in orthorhombic and triclinic forms, respectively. Since the unsubstituted species, 5,5′′-bis(4-biphenylyl)-2,2′:5′,2′′-terthiophene (BP3T), shows unique and superior lasing performance in single crystals, the newly synthesized BP3T-OMe and BP3T-CN have possibilities to show different or improved optoelectronic characteristics. Amplified spontaneous emission (ASE) and optically pumped lasing were observed from both of the single crystals based on their well-shaped crystalline cavity and high group refractive index values of 3.7–5.3 for excellent light confinement. The lasing threshold for the BP3T-OMe crystal was lower than that for the BP3T-CN crystal, which was attributed to their different molecular orientation, standing in the former and inclining in the latter.

## Abstract 2

As new candidates of thiophene/phenylene co-oligomer (TPCO) species, methoxy- or cyano-substituted compounds were synthesized for lasing applications.

## Graphical abstract

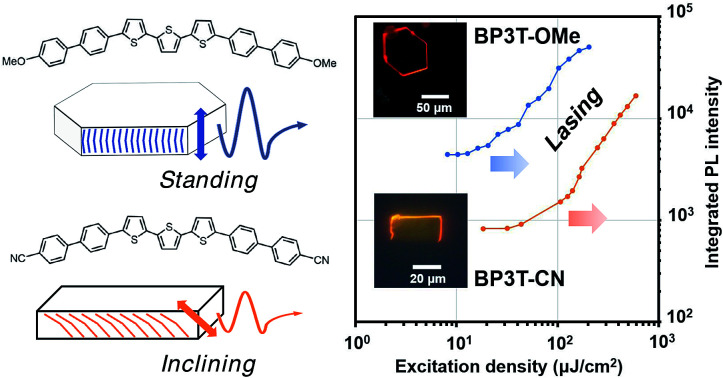

## Introduction

Since the realization of an organic electroluminescence (EL) device composed of an anthracene single crystal reported by M. Pope *et al.* in 1963,^1^ organic light emissive π-conjugated compounds have been paid great attention toward practical applications because of their tunability of light emission wavelengths by changing the molecular structure and their good processability. Afterwards, optically pumped gain-narrowed emissions and lasing have been reported for various organic materials such as π-conjugated polymers,^2^ distyrylbenzene (DSB) derivatives,^3^ thiophene/phenylene co-oligomers (TPCO)^4–10^*etc.* Recently, carbon-bridged oligo-*para*-phenylenevinylene (COPV) derivatives were synthesized to realize low threshold lasing and especially high optoelectronic durabilities.^11,12^ In addition, unique light amplification characteristics based on gain systems using excited state intramolecular proton transfer (ESIPT)^13^ or Förster resonance energy transfer (FRET)^14^ have been realized or proposed to construct efficient gain systems. Of these, gain systems using FRET was also reported toward the realization of electrically pumped lasing, by constructing a host-guest energy transfer system from triplet excited state to singlet excited state.^14^ Beyond optical pumping conditions, electrically pumped gain-narrowed emissions have been recently reported from both organic light emitting diode (OLED) devices^15^ and organic light emitting field-effect transistor (OLEFET) devices.^16^

Among a large number of organic light emissive π-conjugated compounds, thiophene/phenylene co-oligomers (TPCO) which are composed of linearly chained thiophene and phenylene units, are promising materials as active media for lasing under both electrically and optically pumped conditions owing to their high carrier mobilities, high thermal stabilities and high refractive indices.^17–20^ Of these, 5,5′′-bis(4-biphenylyl)-2,2′:5′,2′′-terthiophene (BP3T) has been frequently used as an active medium in OLEFET devices, and electrically pumped gain narrowed emissions were observed due to their ambipolar carrier transport characteristics with high carrier mobilities and high internal quantum efficiency.^21^ Although single crystals of BP3T have been prepared by physical vapor transport (PVT) process, and gain narrowed emissions were successively observed under both optical and electrical pumping, lasing behaviours by self-cavity effect have hardly been investigated due to their curved crystal edges. However, BP3T single crystals show unusual light amplification behaviour, which cannot be explained by the conventional stimulated emission (SE) process. Stimulated resonant Raman scattering (SRRS) was observed from a BP3T single crystal at 12 K,^22^ which indicates an existence of coherent molecular vibrations. The SRRS has also been observed for other TPCO single crystals even at room temperature with emission lines more narrow than that of ASE even without any cavity structure,^22,23^ and their light emission wavelength can be tuned by changing the excitation wavelength.

Moreover, the BP3T single crystal showed pulse-shaped light emission with several tens picoseconds delay relative to the excitation pulse at room temperature.^24,25^ This time-delayed emission in contrast to the SE strongly suggests a cooperative light amplification process similar to superfluorescence or polariton lasing.^25^ As an origin of the delay time, phase relaxation to form a macroscopic ensemble of correlated emitters was expected among highly oriented molecules. According to literature,^26^ BP3T crystallizes in monoclinic form, and the molecular long axis is perpendicular to the crystal basal plane. Since the π–π* transition dipole moments are oriented parallel to the molecular long axis, the emitted light propagates in a transverse magnetic (TM) mode, and the light emission is confined strongly inside a platelet single crystal. This condition of light confinement seems favourable for the formation of aforementioned macroscopic ensemble. Although it's still controversial to interpret the relationships between structure – light amplification characteristics, several reports have shown the fact that amplified spontaneous emission (ASE) or lasing characteristics are critically changed by molecular arrangement depending on the π-electronic interactions,^6^ which is controlled by the modification of constituting molecules in organic crystals.

Here, we report on the synthesis of BP3T derivatives with methoxy- or cyano-groups at the *para*-positions of the terminating ends for further investigations of the aforementioned unique light amplification characteristics. The introduction of substituents leads to various intermolecular interactions, which result in different crystal structures, molecular packings and molecular orientations.

## Results and discussion

**Fig. 1:**
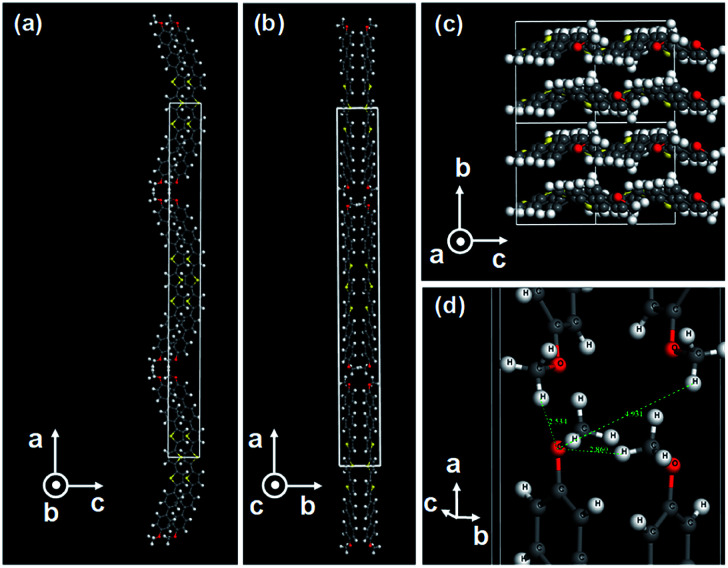
(a–c) Crystal structure of BP3T-OMe with a projection on *ac*-plane, *ab*-plane, and *bc*-plane, respectively. (d) Enlarged structure showing interatomic distances between oxygen and neighboring hydrogen atoms in the methoxy-groups.

Here, we synthesized 5,5′′-bis(4′-methoxy-[1,1′-biphenyl]-4-yl)-2,2′:5′,2′′-terthiophene (BP3T-OMe) and 4′,4′′′-([2,2′:5′,2′′-terthiophene]-5,5′′-diyl)bis(([1,1′-biphenyl]-4-carbonitrile)) (BP3T-CN), following a previously published report.^26^ XRD measurements revealed crystal structures for both single crystals of BP3T-OMe (CCDC-1991924) and BP3T-CN (CCDC-1991925).† BP3T-OMe crystallized in an orthorhombic form (*a* = 65.19 Å, *b* = 7.43 Å, *c* = 5.80 Å, space group: *Cmc*2_1_, *Z* value: 4, cell volume: 2808 Å^3^). Same as in the case of BP3T, the π-electronic transition dipole moment is oriented parallel to the molecular long axis. As shown in Fig. 1(a)–(c), BP3T-OMe takes perfectly perpendicular molecular orientation to the crystal bottom plane (*bc*-plane), which is ideal to confine the cavity photon inside a single crystal with in-plane direction. Although the orientation of the molecular long axis of BP3T is also perpendicular to the crystal bottom plane (*ab*-plane), however, it's a little inclined with a tilting angle of 2.6°.^26^ Therefore, the orientation of BP3T-OMe seems more favourable to confine photons in a cavity consisting of a single crystal, comparing to the case of BP3T. The origin of the perpendicularly aligned molecular orientation in the BP3T-OMe single crystal can be attributed to the H-bonding interaction between methoxy-groups of adjacent molecules as shown in Fig. 1(d). In a previous report about other methoxy-substituted TPCO compounds, 2,5-bis(4′-methoxybiphenyl-4-yl)thiophene (BP1T-OMe) showed the same perpendicular orientation as a consequence of the hydrogen bonding between the methoxy-groups.

**Fig. 2:**
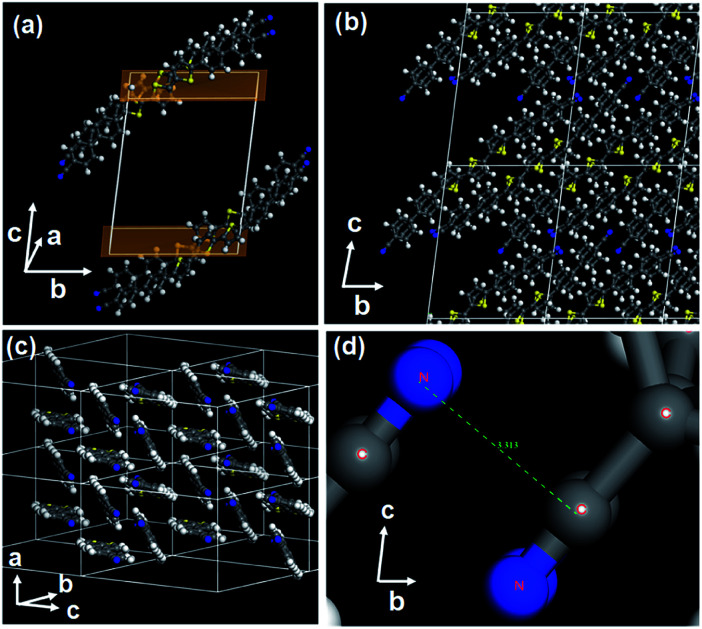
(a) Crystal structure of BP3T-CN showing a tilted orientation of the long molecular axes relative to the crystal basal plane. (b and c) Crystal structure of BP3T-CN showing the molecular packing in multiple unit cells. (d) Enlarged structure showing the interatomic interaction between adjacent cyano-groups.

On the other hand, BP3T-CN crystallized in a triclinic form (*a* = 9.49 Å, *b* = 14.92 Å, *c* = 19.58 Å, *α* = 82.73°, *β* = 87.51°, *γ* = 89.92°, space group: *P*1̄, *Z* value: 4, cell volume: 2746 Å^3^). The molecular axis of BP3T-CN is tilted by about 45° against the bottom plane (*ab*-plane) as shown in Fig. 2(a) and (b). An origin of the obliquely lying orientation is attributed to the electrostatic interaction between cyano-substituents of neighbouring molecules, as revealed in a previous report for 2,5-bis(4′-cyanobiphenyl-4-yl)thiophene (BP1T-CN).^10^ The cyano-groups are oppositely oriented at the intermolecular interface, therefore, the C(*δ*+)–N(*δ*−) interaction is formed between the adjacent molecular terminals as shown in Fig. 2(d). These lying molecular orientation is suitable for the realization of vertical cavity surface emitting lasers (VCSELs) using distributed Bragg reflector (DBR) mirrors since the tilted transition dipole moments allow sufficient emission components to the crystal surface direction.^28,29^ Moreover, organic polariton lasers have recently been investigated using VCSEL structures to realize low threshold lasing devices. Actually, gain-narrowed emissions from VCSEL structures were observed using cyano-substituted TPCOs as active media in VCSELs.^28,29^

**Fig. 3:**
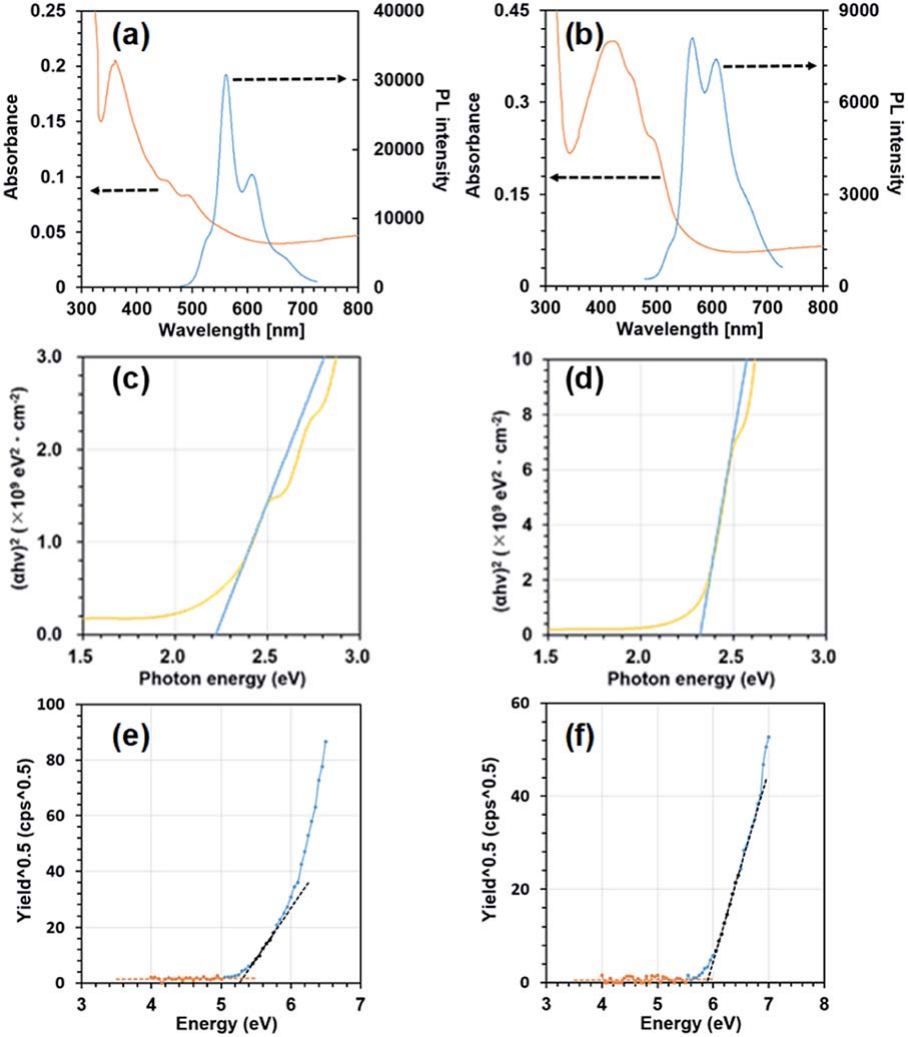
Absorption and PL spectra for BP3T-OMe (a) and BP3T-CN (b), (*αhν*)^2^*vs.* energy plots for estimation of band-gap values of BP3T-OMe (c) and BP3T-CN (d), (photon yield)^0.5^*vs.* energy plots for estimation of HOMO values for BP3T-OMe (e) and BP3T-CN (f).

Next, we measured absorption and photoluminescence (PL) spectra using vapour deposited thin films as shown in Fig. 3(a) and (b). Fig. 3(c) and (d) show plots of (*αhν*)^2^ as a function of photon energy where *α* and *h* and *ν* represent the absorption coefficient, Plank's constant and the frequency, respectively. The values of *α* were obtained from the respective absorbance divided by sample thickness. The frontier orbital gap value (*E*_g_) was obtained by extrapolating the linear slope of (*αhν*)2 to the zero absorption coefficient (*α* = 0). The obtained *E*_g_ values were 2.22 eV and 2.32 eV for BP3T-OMe and BP3T-CN, respectively. Fig. 3(e) and (f) show plots of (*photon yield*)^0.5^ as a function of incident photon energy in the measurements of photoelectron spectroscopy in air (PESA). The highest occupied molecular orbital (HOMO) levels estimated from the threshold photon energy in Fig. 3(e) and (f) were −5.31 and −5.91 eV for BP3T-OMe and BP3T-CN, respectively. The lowest unoccupied molecular orbital (LUMO) levels calculated by the HOMO levels and aforementioned frontier orbital gap energy values were −3.09 and −3.59 eV for BP3T-OMe and BP3T-CN, respectively. The HOMO and LUMO levels of BP3T estimated by the same procedures were −5.78 eV and −3.40 eV, respectively. These results show that the methoxy- and cyano-group act as electron donating and electron withdrawing substituents, therefore, p-type and n-type semiconducting characteristics can be expected in BP3T-OMe and BP3T-CN, respectively.

**Fig. 4:**
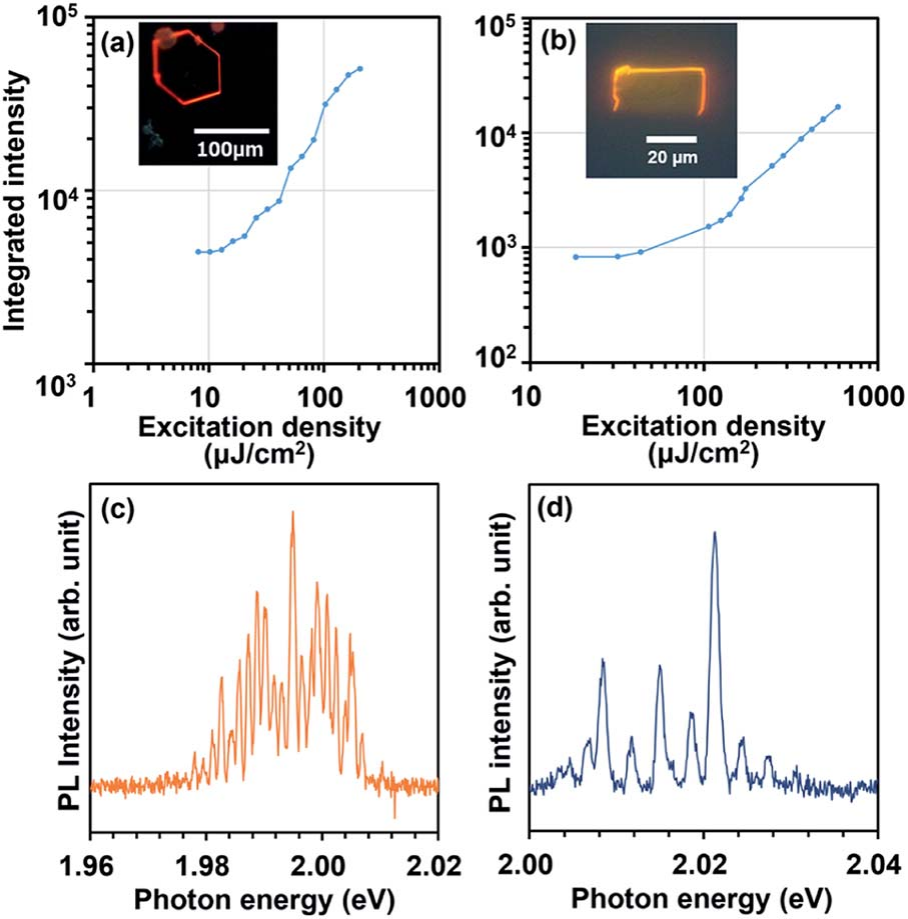
(a) Excitation density dependence of integrated PL intensity for 0–2 band taken from single crystals of BP3T-OMe (a) and BP3T-CN (b) with their fluorescence image in the inset. PL spectra taken from single crystals of BP3T-OMe at 162 μJ cm^−2^ (c) and BP3T-CN at 602 μJ cm^−2^ (d) showing longitudinal multi-mode lasing.

Lasing characteristics of their derivative crystals were investigated under optical pumping using a Nd:YAG pulsed laser as an excitation source. As the excitation density is elevated, the emission intensity was nonlinearly increased at a certain threshold both for BP3T-OMe and BP3T-CN crystals, as shown in Fig. 4(a) and (b). The threshold excitation density was estimated to be 17 μJ cm^−2^ for BP3T-OMe and 152 μJ cm^−2^ for BP3T-CN, respectively. The threshold for the BP3T-OMe single crystal was lower than that of the BP3T-CN single crystal, which can be attributed to their different molecular orientation, as discussed in Results and discussion part. The perpendicular orientation to the crystal bottom plane in the BP3T-OMe single crystal is favourable for the efficient stimulated emission along the planar cavity compared to the inclining orientation in the BP3T-CN single crystal. As shown in Fig. 4(c) and (d), longitudinal multi-mode lasing spectra were observed both for BP3T-OMe and BP3T-CN single crystal, owing to the self-cavity effect in a well-shaped single crystal. Group refractive index (*n*_g_) values were calculated using an equation of *n*_g_ = 1/2*LΔλ*^−1^, where *L* is the cavity length estimated by the fluorescence micrograph shown in Fig. 4(a) and (b), *Δλ*^−1^ is the mode intervals at each peaks of longitudinal multi-mode lasing spectra. The calculated *n*_g_ value was 4.8 for BP3T-OMe while that of a BP3T-CN single crystal was 3.7–5.3. As both single crystals of BP3T-OMe and BP3T-CN showed lasing performances based on those high group refractive indices as similarly reported for other TPCO single crystals, these newly synthesized BP3T derivatives are strongly expected as promising organic lasing media.

## Experiments

### Synthesis

**Scheme 1:**
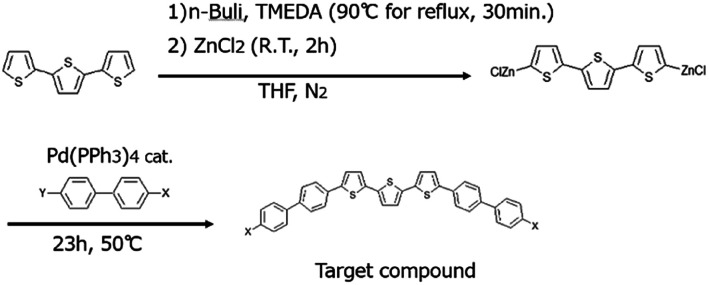
Synthesis protocol of BP3T-OMe and BP3T-CN where X = methoxy, Y = bromide for BP3T-OMe, and X = cyano, Y = iodide for BP3T-CN.

To the best of our knowledge, only BP3T-OMe has been synthesized *via* coupling reactions, so far.^30^ Here we synthesized both BP3T-OMe and BP3T-CN *via* a Negishi coupling reaction as shown in Scheme 1.

#### Synthesis of BP3T-OMe

4-Bromo-4′-hydroxybiphenyl (5.004 g, 20.09 mmol), potassium carbonate (13.873 g, 100.38 mmol) and anhydrous acetone (50 mL) were put into a two-neck flask. To this solution, iodomethane (1.6 mL) was added and then heated for 24 hours under reflux. Then, all of the solution was gathered in a beaker and water was added until all of the solid was dissolved. Next, the solution was put into a separation funnel, and extracted 3 times using 100–300 mL dichloromethane. Then, the dichloromethane phase was gathered in a 1 neck flask, and magnesium sulfate was added to remove residual water. After filtrating this solution, all of the solvent was removed, and the remaining solid was recrystallized 3 times using acetone as highly soluble solvent and *n*-hexane as poor soluble solvent, yielding 4-bromo-4′-methoxybiphenyl (4.21 g, 16.0 mmol, 80%).

2,2′:5′,2′′-Terthiophene (681 mg, 2.74 mmol) was mixed with anhydrous THF (10 mL) and *N*,*N*,*N*′,*N*′-tetramethylethylenediamine (1.00 mL, 6.68 mmol). To this solution, 4 mL of 1.6 M *n*-butyllithium in cyclohexane was added dropwise at 0 °C and the reaction mixture was refluxed for 30 minutes and successively cooled down to −10 °C. Anhydrous zinc chloride (1.233 g, 9.045 mmol) dispersed in anhydrous tetrahydrofuran (15 mL) was added and the reaction solution was kept at 25 °C for 2 hours. Tetrakis(triphenylphosphine)-palladium(0) (106.5 mg, 0.09220 mmol) was immediately added and subsequently 4-bromo-4′-methoxybiphenyl (1.743 g, 6.624 mmol) dispersed in anhydrous tetrahydrofuran (5 mL) was added. The reaction mixture was warmed to 50 °C and kept for 23 hours to yield a precipitate. The precipitate was washed with 50 mL THF, 50 mL H_2_O, 10 mL 10% HCl, 50 mL H_2_O, 50 mL acetone and 250 mL hot 1,2,4-trichlorobenzene, yielding 377 mg (0.615 mmol, 22%).

#### Synthesis of BP3T-CN

4-Biphenylcarbonitrile (5.0037 g, 27.919 mmol) was mixed with acetic acid (13.7 mL) and water (2.8 mL) and 98% sulfuric acid (0.42 mL) in a one-neck flask. Iodine (3.646 g, 14.37 mmol) and periodic acid (1.405 g, 6.164 mmol) were added. After that, the mixture was warmed to 90 °C for 24 hours. During this time, the solution color changed from colorless to red and a precipitate formed. The precipitate was washed by water and methanol alternately 3 times, and dissolved in chloroform (100 mL). This solution was washed with a saturated aqueous sodium chloride solution and dried over anhydrous magnesium sulfate. After evaporating chloroform, the remaining solid was dissolved in hot ethyl acetate (50 mL). Methanol (25 mL) was added and cooled down to 5 °C for crystallization. The precipitates were isolated by filtration and dried to give yellow crystals of 5.199 g (17.04 mmol, 61%) of 4′-iodo-biphenyl-4-carbonitrile, which was identified by ^1^H NMR (solvent: deuteriochloroform) and showed the same chemical shift and proton integral values as reported in the literature.^27^ Using this obtained compound, BP3T-CN was synthesized according to the reaction Scheme 1. 2,2′:5′,2′′-Terthiophene (671 mg, 2.701 mmol) was mixed with anhydrous THF (10 mL) and *N*,*N*,*N*′,*N*′-tetramethylethylenediamine (1.00 mL, 6.68 mmol). To this solution, 4 mL of 1.6 M *n*-butyllithium in cyclohexane was added dropwise at 0 °C and the reaction mixture was heated under reflux for 30 minutes and successively cooled down to −10 °C. Anhydrous zinc chloride (1.335 g, 9.793 mmol) dispersed in anhydrous tetrahydrofuran (15 mL) was added and the reaction solution was kept at 25 °C for 2 hours. Tetrakis(triphenylphosphine)-palladium(0) (0.105 g, 0.0909 mmol) was immediately added and subsequently 4′-iodo-biphenyl-4-carbonitrile (2.043 g, 6.696 mmol) dispersed in anhydrous tetrahydrofuran (5 mL) were added. The reaction mixture was warmed to 50 °C for 23 hours to yield a precipitate. This precipitate was filtered-off and washed with 50 mL THF, 50 mL H_2_O, 10 mL 10% HCl, 50 mL H_2_O, 50 mL acetone, and 250 mL hot 1,2,4-trichlorobenzene, and further purified by sublimation under a vacuum at 300 °C. The solid remained in the heating zone was collected as the purified target compound, yielding 612 mg (1.02 mmol, 38%).

Due to the low solubility in any solvents for BP3T-OMe and BP3T-CN, identification by ^1^H-NMR or ^13^C-NMR were unavailable. However, both of the target compounds were identified by MALDI-TOF-MS, found [M] = 612 and 602, for BP3T-OMe and BP3T-CN respectively. Almost all of TPCO series were identified by mass spectroscopy instead of using NMR spectroscopy in previous reports.^27,30^

### Preparation of single crystals and vapour deposited thin films

Single crystals for X-ray diffraction measurements were prepared *via* a modified sublimation process. Purified powder of BP3T-OMe or BP3T-CN was put in a quartz crucible, and the molecules were sublimed and collected into another quartz crucible facing each other in a vacuum chamber (2 × 10^−4^ to 4 × 10^−4^ Pa). Single crystals of BP3T-CN for PL measurements were prepared by the same process used in X-ray diffraction measurements. Single crystals of BP3T-OMe for PL measurements were prepared *via* a solution growth process. First, a saturated BP3T-OMe solution was prepared at 150 °C using 1,2,4-trichlorobenzene as solvent. The solution was heated to 190 °C, then, successively cooled down to 40 °C for 18 hours. Thin films of BP3T-OMe and BP3T-CN for optical and photoelectron spectroscopy measurements were prepared by vapour deposition onto glass substrates. Purified powder of BP3T-OMe or BP3T-CN was evaporated from a resistively heated a quartz crucible under a vacuum condition (2 × 10^−4^ to 4 × 10^−4^ Pa). Their deposition rate and film thickness were monitored by a quartz crystal microbalance.

### Optical characterizations

Absorption and PL spectra were measured for the vapour-deposited thin films of BP3T-OMe and BP3T-CN using an ultraviolet-visible (UV-vis) spectrometer (JASCO V-530). The work functions of BP3T-OMe and BP3T-CN were measured by photo-electron spectroscopy in air (PESA, Riken Keiki AC-3) at the vapour-deposited thin films on ITO/glass substrates. Fluorescence images as well as PL spectra of single-crystal samples were measured using a fluorescence microscope (Olympus BX-51) equipped with a charge coupled device (CCD) spectrometer (Hamamatsu PMA-50) through a 10× objective lens.

### Observation of optically pumped lasing

Single crystals of BP3T-OMe grown *via* the solution process were transferred onto glass substrates by immersing the substrates into the solution, and then dried at atmosphere. Single-crystals of BP3T-CN were transferred onto glass substrates using a metal needle. Their fluorescence images were taken under ultraviolet excitation. Optically pumped PL measurements were performed using a Nd:YAG pulsed laser (*λ*_ex_ = 355 nm, pulse frequency: 1.2 kHz, pulse duration: 1.1 ns) at room temperature under ambient conditions. The excitation beam shaped into a stripe of 0.24 × 0.04 cm^2^ through a cylindrical lens was focused on the single crystal surface with an incident angle of ∼45°. Photoemission radiated from the single-crystal edge was detected using a CCD spectrometer (Hamamatsu PMA-50) through an optical fiber.

## Conclusions

BP3T-OMe and BP3T-CN were successfully synthesized and their crystal structures were identified. BP3T-OMe and BP3T-CN crystallized in orthorhombic and triclinic forms, respectively. The molecular orientation of BP3T-OMe is perfectly perpendicular to the crystal basal plane, suggesting more efficient light confinement in the single-crystal cavity in a TM mode comparing to that of BP3T single crystals with slightly inclined orientation. On the other hand, a molecular orientation of BP3T-CN was oblique to the crystal basal plane, which is suitable for surface-emitting devices. The HOMO and LUMO levels of BP3T-OMe and BP3T-CN estimated by absorption and photoelectron spectroscopies revealed that the methoxy-group and cyano-groups act as electron-donating and electron-withdrawing groups, leading to expected p- and n-type semiconduction, respectively. Single crystals of BP3T-OMe and BP3T-CN showed optically pumped lasing with especially high group refractive index values, indicating the possibility to serve as good active media for organic lasers, especially in OLED structures due to their p- and n-type characteristics. Furthermore, the lasing threshold for the BP3T-OMe crystal was lower than that for the BP3T-CN crystal, which was attributed to their different molecular orientation, standing in the former and inclining in the latter.

## Conflicts of interest

There are no conflicts to declare.

## Supplementary Material

RA-010-D0RA04742B-s001
